# Accessory Nerve Schwannomas Presenting with Hypoglossal Nerve Palsy: A Narrative Review with an Illustrative Case

**DOI:** 10.3390/life16040655

**Published:** 2026-04-13

**Authors:** Gen Futamura, Ryokichi Yagi, Masao Fukumura, Moeko Tani, Hideki Kashiwagi, Yuichiro Tsuji, Ryo Hiramatu, Masahiro Kameda, Naosuke Nonoguchi, Motomasa Furuse, Shinji Kawabata, Toshihiro Takami, Masahiko Wanibuchi

**Affiliations:** Department of Neurosurgery, Osaka Medical and Pharmaceutical University, Takatsuki 569-8686, Japan; ryokichi.yagi@ompu.ac.jp (R.Y.); masao.fukumura@ompu.ac.jp (M.F.); moeko.tani@ompu.ac.jp (M.T.); hideki.kashiwagi@ompu.ac.jp (H.K.); yuuichirou.tsuji@ompu.ac.jp (Y.T.); ryo.hiramatsu@ompu.ac.jp (R.H.); masahiro.kameda@ompu.ac.jp (M.K.); naosuke.nonoguchi@ompu.ac.jp (N.N.); motomasa.furuse@ompu.ac.jp (M.F.); shinji.kawabata@ompu.ac.jp (S.K.); toshihiro.takami@ompu.ac.jp (T.T.); wanibuchi@ompu.ac.jp (M.W.)

**Keywords:** accessory nerve schwannoma, jugular foramen schwannoma, hypoglossal nerve palsy, skull base tumor, diagnostic pitfalls, differential diagnosis

## Abstract

**Background:** Intracranial accessory nerve schwannomas involving the jugular foramen are rare tumors with heterogeneous clinical presentations. Although lower cranial nerve dysfunction is common, hypoglossal nerve palsy is uncommon and may obscure identification of the nerve of origin. **Methods:** A narrative review of the literature was conducted to identify reported cases from 1961 to December 2025. Clinical manifestations were categorized as initial and preoperative symptoms, and their temporal evolution was analyzed according to tumor location. Imaging findings, surgical management, and neurological outcomes were reviewed. An illustrative case with hypoglossal nerve palsy was included. **Results:** A total of 58 cases, including the present case, were identified. According to the Julow classification, 38 tumors were intracisternal and 20 were intrajugular. Intracisternal tumors predominantly caused posterior fossa compression symptoms, whereas intrajugular tumors more frequently showed lower cranial nerve dysfunction. Hypoglossal nerve palsy was observed in seven cases, including three as the initial symptom, and occurred mainly in intrajugular tumors. Imaging commonly demonstrated jugular foramen enlargement and, in selected cases, continuity with enlargement of the extracranial hypoglossal canal. Surgical treatment was associated with improvement or stabilization of hypoglossal nerve function in all reported cases. **Conclusions:** Accessory nerve schwannomas may occasionally present with hypoglossal nerve palsy, most likely due to secondary compression. Careful assessment of symptom progression and skull base imaging may improve preoperative diagnosis and surgical planning.

## 1. Introduction

Schwannomas are benign nerve sheath tumors that account for approximately 8% of all primary intracranial tumors [1]. Most schwannomas arise from sensory cranial nerves, most commonly the vestibular nerve, whereas those originating from the lower cranial nerves are relatively uncommon. Among these, jugular foramen schwannomas—arising from the glossopharyngeal, vagus, or accessory nerves—account for approximately 2% of intracranial schwannomas [1,2,3].

Accessory nerve schwannomas are considered extremely rare, representing the third most common subtype among jugular foramen schwannomas after those arising from the glossopharyngeal and vagus nerves [4,5,6,7,8]. This rarity is thought to reflect the fact that intracranial schwannomas typically originate from sensory nerves, whereas tumors arising from purely motor nerves, such as the accessory nerve, are uncommon [1,9]. Consequently, accessory nerve schwannomas have been reported far less frequently than schwannomas of other lower cranial nerves, and their clinical characteristics remain incompletely understood. Previous studies have shown that these tumors can present with diverse neurological symptoms depending on their anatomical location, growth pattern, and relationship to adjacent neurovascular structures [7,9,10,11,12,13,14].

Hypoglossal nerve palsy is a well-recognized presenting feature of hypoglossal schwannomas, occurring in approximately 66.7–92.9% of cases and typically manifesting as tongue deviation, atrophy, and dysarthria [6,15,16,17,18]. In contrast, hypoglossal nerve palsy is rarely observed in jugular foramen schwannomas, including those arising from the accessory nerve [19,20]. When hypoglossal nerve palsy does occur in this setting, it may obscure identification of the true nerve of origin and create a significant diagnostic pitfall. This discrepancy between clinical presentation and tumor origin poses an important challenge for accurate preoperative diagnosis and surgical planning.

With advances in skull base imaging and microsurgical techniques, a detailed assessment of tumor extension patterns and foraminal involvement has become increasingly important for differentiating among lower cranial nerve schwannomas. At the same time, the literature on accessory nerve schwannomas has accumulated gradually over several decades, but remains composed largely of isolated case reports and small series. Consequently, the available evidence is fragmented, and comprehensive reviews focusing specifically on accessory nerve schwannomas presenting with hypoglossal nerve palsy remain limited.

In this context, a clearer synthesis of the published literature is needed not only to summarize the known clinical spectrum of this rare entity but also to highlight its diagnostic pitfalls and unresolved questions. Therefore, the aim of this narrative review is to summarize the existing literature on intracranial accessory nerve schwannomas, with particular emphasis on cases presenting with hypoglossal nerve palsy. We review their epidemiology, classification, clinical presentation, imaging characteristics, management strategies, and postoperative neurological outcomes. In addition, an illustrative case is presented to highlight the anatomical basis of this diagnostic challenge and to underscore the importance of meticulous preoperative imaging assessment in patients with lower cranial nerve dysfunction.

## 2. Methods

### Literature Search Strategy

To identify previously reported cases of intracranial accessory nerve schwannomas, a narrative review of the literature was conducted. A systematic search of the PubMed database was performed using the same search terms employed by Yan et al. [14], covering the period from 1961 to December 2025. The final search was completed on 11 February 2026. The following keywords and their combinations were used: “accessory nerve schwannoma,” “spinal accessory nerve schwannoma,” “accessory nerve neurinoma,” “spinal accessory nerve neurinoma,” “accessory nerve sheath tumor,” and “spinal accessory nerve sheath tumor.”

The database search identified 196 records. After removal of duplicate and clearly irrelevant records, 40 articles underwent title and abstract screening. Full-text assessment was subsequently performed for these 40 articles, and 30 were excluded for the following reasons: extracranial-only location (*n* = 11), spinal canal-only location (*n* = 1), and non-accessory nerve tumors or insufficient anatomical information (*n* = 18). In addition, manual screening of the reference lists of eligible articles identified 39 additional publications. Ultimately, 57 previously reported cases from 49 articles were included in the review, and together with the present case, a total of 58 cases were analyzed.

Eligible studies included original case reports and case series describing accessory nerve schwannomas with intracranial involvement and/or extension to the jugular foramen. Studies reporting tumors confined exclusively to extracranial locations or limited to the spinal canal were excluded. Only articles with an English abstract were considered. Cases associated with neurofibromatosis (von Recklinghausen disease) were excluded whenever possible in order to focus on sporadic accessory nerve schwannomas and to minimize potential confounding related to tumor multiplicity and genetic background.

For each eligible case, the following variables were extracted when available: patient age and sex, initial symptoms, tumor location and classification, tumor volume, surgical approach or adjuvant therapy, preoperative and postoperative cranial nerve dysfunction, and neurological outcome. The nerve of origin was identified according to the descriptions provided in the original reports. In most cases, this determination was based primarily on intraoperative findings. Among the reviewed studies, only Yoo et al. [21] explicitly stated that the nerve of origin was determined either by intraoperative findings alone or by inference from a combination of intraoperative and radiological findings. When tumor volume was not explicitly reported, it was estimated from the available imaging information according to the method described in the footnotes of the tables.

Because the available literature consisted predominantly of isolated case reports and small case series, no formal study-quality assessment tool was applied. Likewise, this study was not designed as a PRISMA-based systematic review, as the rarity of the condition and the heterogeneity of the source reports limited the feasibility of formal risk-of-bias assessment and quantitative synthesis. Instead, our aim was to provide a comprehensive descriptive and hypothesis-generating review of the available evidence.

To complement the literature review and to illustrate the diagnostic challenges encountered when accessory nerve schwannomas present with hypoglossal nerve palsy, an illustrative case from our institution was included.

## 3. Results

### 3.1. Overview of Reported Cases

A literature search identified a total of 58 cases of accessory nerve schwannomas with intracranial involvement or extension to the jugular foramen., including the present case (Table 1) [1,3,6,7,10,11,12,13,14,19,20,21,22,23,24,25,26,27,28,29,30,31,32,33,34,35,36,37,38,39,40,41,42,43,44,45,46,47,48,49,50,51,52,53,54,55,56,57]. Among the 58 cases, including the present case, 26 patients were female and 27 were male, while sex was not reported in five cases. The median age at symptom onset was 47 years (range, 18–76 years).

### 3.2. Tumor Location and Classification

Tumors were classified according to both the Julow classification [11] and the jugular foramen schwannoma (JFS) classification proposed by Kaye and Pellet [58,59].

The Julow classification, which is widely applied to accessory nerve schwannomas, categorizes tumors based on anatomical location into intracisternal and intrajugular types. In contrast, the JFS classification categorizes tumors into four types: Type A (intracranial), Type B (primarily within the jugular foramen), Type C (primarily extracranial), and Type D (dumbbell-shaped tumors extending both intra- and extracranially).

According to the Julow classification, 38 tumors were classified as the intracisternal type and 20 as the intrajugular type, indicating that intracisternal tumors were reported more frequently. Under the JFS classification, Type B and Type D tumors predominated among intrajugular lesions, whereas nearly all intracisternal tumors were classified as Type A.

### 3.3. Clinical Manifestations and Cranial Nerve Dysfunction

For analysis of clinical manifestations, symptoms were categorized as initial symptoms and preoperative symptoms. Initial symptoms were defined as those clearly described in the literature as the first symptoms recognized by the patient or, in cases in which only a single symptom was reported, that symptom was regarded as the initial symptom. Symptoms for which the timing of onset was not clearly specified, as well as symptoms that newly developed between the onset of initial symptoms and surgical intervention, were classified as preoperative symptoms.

Because the clinical manifestations reported by Tsukamoto et al. [9] were considered to be primarily attributable to a concomitant giant meningioma rather than to the accessory nerve schwannoma itself, that case was excluded from the symptom-frequency analyses. Accordingly, the analyses presented in Table 2, Table 3 and Table 4 were performed in the remaining 57 cases.

#### 3.3.1. Frequency of Initial Symptoms (Table 2)

Analysis of initial symptoms revealed that headache, nausea/vomiting, gait ataxia, and hoarseness were the most frequently reported manifestations. With the exception of hoarseness, other lower cranial nerve-related symptoms were relatively uncommon at initial presentation.

Symptoms suggestive of hypoglossal nerve palsy were observed in only three cases (5.3%), including the present case.

When stratified by tumor location, intracisternal tumors predominantly presented with symptoms related to cerebellar or brainstem compression, including headache, nausea/vomiting, and gait ataxia. In contrast, intrajugular tumors more frequently presented with lower cranial nerve-related symptoms, such as hoarseness, shoulder weakness, dysphagia, and tongue deviation.

#### 3.3.2. Combined Analysis of Initial and Preoperative Symptoms (Table 3)

When initial and preoperative symptoms were analyzed together, headache was the most frequent clinical manifestation, followed by hoarseness, gait ataxia, nausea/vomiting, and sternocleidomastoid and/or trapezius muscle atrophy.

Hearing loss, which is commonly observed in jugular foramen schwannoma [1,4,8,45,58,59], was relatively uncommon in accessory nerve schwannomas.

Clear differences in symptom distribution were observed according to tumor location. In the intracisternal type, symptoms related to posterior fossa compression—such as headache, nausea/vomiting, and gait ataxia —were predominant. In contrast, the intrajugular type showed a higher frequency of lower cranial nerve dysfunction, including hoarseness, sternocleidomastoid and/or trapezius muscle atrophy, shoulder weakness, dysphagia, hypoglossal nerve–related symptoms, and hearing loss.

#### 3.3.3. Temporal Evolution of Cranial Nerve Symptoms According to Tumor Location

Table 4 summarizes the temporal evolution of cranial nerve–related symptoms at initial presentation and during the preoperative course according to tumor location.

In intrajugular tumors (*n* = 20), lower cranial nerve–related symptoms were already present at initial presentation, most commonly hoarseness (cranial nerve: CN X, *n* = 6), followed by shoulder weakness (CN XI, *n* = 4) and tongue deviation or atrophy (CN XII, *n* = 3). During the preoperative course, these symptoms increased in frequency, particularly dysphagia (CN IX, from *n* = 2 to *n* = 9) and hearing loss (CN VIII, from *n* = 1 to *n* = 5). Sternocleidomastoid and/or trapezius muscle atrophy (CN XI) developed preoperatively in eight cases, despite being absent at initial presentation.

In contrast, intracisternal tumors (*n* = 37) showed few lower cranial nerve symptoms at onset. During follow-up, only a limited number of cases developed hearing loss (CN VIII, *n* = 2), dysphagia (CN IX, *n* = 1), or shoulder weakness (CN XI, *n* = 4), and hypoglossal nerve-related symptoms remained uncommon.

Overall (*n* = 57), the most frequent symptoms were hoarseness (26.3%), shoulder weakness (21.1%), sternocleidomastoid and/or trapezius muscle atrophy (21.1%), and dysphagia (17.5%). Hearing loss was observed in 7 cases (12.3%), whereas hypoglossal nerve palsy was observed in 7 cases (12.3%) and dysarthria was rare (1 case, 1.8%).

### 3.4. Imaging Findings

On magnetic resonance imaging (MRI), accessory nerve schwannomas typically appeared as hypo- to isointense lesions on T1-weighted images and as heterogeneously hyperintense lesions on T2-weighted images. Contrast-enhanced T1-weighted images demonstrated either homogeneous or mildly heterogeneous enhancement [1,3,10,14,19,35,37,38,39,40,42,45,46,49,52,56].

On computed tomography (CT), smooth enlargement of the jugular foramen with associated bone scalloping and sclerotic margins is frequently observed. In many cases, the cortical bone remains preserved. These radiological features have been reported to be important in the differential diagnosis among schwannomas, meningiomas, and glomus jugulare tumors [1,3,10,19,20,21,35,45,47,58].

In cases presenting with hypoglossal nerve palsy, the imaging feature most suggestive of accessory nerve origin was enlargement of the jugular foramen, sometimes accompanied by enlargement of the extracranial portion of the hypoglossal canal. In selected cases, including the present illustrative case, continuity between the expanded jugular foramen and the extracranial hypoglossal canal was identified, suggesting secondary involvement of the hypoglossal canal rather than a primary hypoglossal canal tumor [41]. However, this finding was not documented uniformly in all reported cases with hypoglossal nerve palsy.

Detailed assessment of such osseous involvement can be achieved using thin-slice computed tomography protocols reported in the literature, with slice thicknesses ranging from 0.5 to 3 mm, particularly when combined with bone window settings and multiplanar reconstruction in axial, coronal, and sagittal planes [3,45,47].

### 3.5. Accessory Nerve Schwannomas Presenting with Hypoglossal Nerve Palsy (Table 5)

Hypoglossal nerve palsy as an initial presenting symptom was identified in three cases, including the present case. When cases in which hypoglossal nerve palsy developed during the preoperative course were also included, a total of seven cases exhibited hypoglossal nerve palsy prior to surgery.

Among the three cases with hypoglossal nerve palsy as the initial symptom, two were classified as the intrajugular type and one as the intracisternal type according to the Julow classification. Under the JFS classification, one tumor was classified as Type A and two as Type B. The intrajugular tumors extended from the jugular foramen toward the extracranial space, whereas the intracisternal tumor was located near the foramen of Luschka and extended toward the foramen magnum.

In the additional four cases in which hypoglossal nerve palsy developed during the preoperative course, all tumors were predominantly intrajugular. In these cases, hypoglossal nerve palsy appeared after the onset of other cranial nerve symptoms or in association with tumor progression.

All seven patients underwent surgical treatment tailored to tumor location and extent. Postoperative neurological outcomes were available for all cases. Hypoglossal nerve function improved in six cases and remained unchanged in one case, and no patient experienced postoperative worsening of hypoglossal nerve palsy. Postoperative deterioration of accessory nerve function was reported in one case, whereas other cranial nerve functions were preserved or improved.

**Table 5:** Accessory Nerve Schwannomas Presenting with Hypoglossal Nerve Palsy.

Authors & Year	Sex	Age	Symptoms				location	Tumor Volume *	Surgical Approach	Extent of Resection	Preoperative CN Deficit	New Postoperative CN Deficit	Postoperative CN Recovery
			Initial	Preoperative	Julow et al. [11] Classification	JFS Classification	Details						
Pluchino et al., 1975 [23]	female	42	headache	absent corneal reflexes, hearing loss, hoarseness, tongue atrophy, gait ataxia	Intrajugular type	Type D	Involving the medial aspect of the petrous bone and the occipital condyle	-	Lateral suboccipital approach	GTR	V, VIII, X, XII	None	V, VIII, X, XII
Pou-Serradell et al., 1978 [24]	male	56	hoarseness	hearing loss, dysphagia, shoulder weakness, shoulder pain, sternocleidomastoid and trapezius muscles atrophy, tongue atrophy	Intrajugular type	-	-	-	Lateral suboccipital approach	GTR	VIII, IX, X, XI, XII	None	VIII, IX, X, XII
Saydam et al., 1997 [41]	female	52	hoarseness, shoulder weakness	dysphagia, trapezius atrophy, tongue deviation	Intrajugular type	Type C	Jugular foramen with extracranial extension	-	Transcervical-transmandibular approach	GTR	IX, X, XI, XII	None	IX, X, XI, XII
Wilson et al., 2005 [45]	-	-	tongue deviation	None	Intrajugular type	Type B	Jugular foramen with extracranial extension	>13.5 **	Transcondylar approach	-	XII	XI	XII
Wilson et al., 2005 [45]	-	-	-	dysphagia, hoarseness, tongue deviation	Intrajugular type	Type D	Jugular foramen tumor extending into the posterior fossa and extracranial space	>13.5 **	Infratemporal approach with partial VII mobilization	-	IX, X, XII	None	None
Richard et al., 2017 [13]	female	52	dizziness, hoarseness, tongue deviation	None	Intracisternal type	Type A	Foramen of Luschka extending to foramen magnum	21.9	Lateral suboccipital approach with C1 laminectomy	GTR	X, XII	None	XII
Present case	female	62	tongue deviation and atrophy, dysarthria	dysphagia, hoarseness, trapezius muscle atrophy	Intrajugular type	Type B	Jugular foramen with extracranial extension (partly into the hypoglossal canal)	10.3	Transcondylar approach	RCIR	IX, X, XI, XII	None	IX, X, XII

- indicates data not reported in the original publication. * When all three tumor dimensions (a, b, and c) were available, tumor volume was estimated using the ellipsoid formula (a × b × c/2, cm^3^). ** In the report by Wilson et al., tumor volume was not explicitly described; however, the minimum reported tumor diameter was 3 cm. Assuming a spherical tumor with a diameter of 3 cm, the estimated tumor volume was at least 13.5 cm^3^. CN, cranial nerve; GTR, gross total resection; JFS, jugular foramen schwannoma; RCIR, radiographically complete intracapsular resection.

### 3.6. Illustrative Case

**Figure 1:**
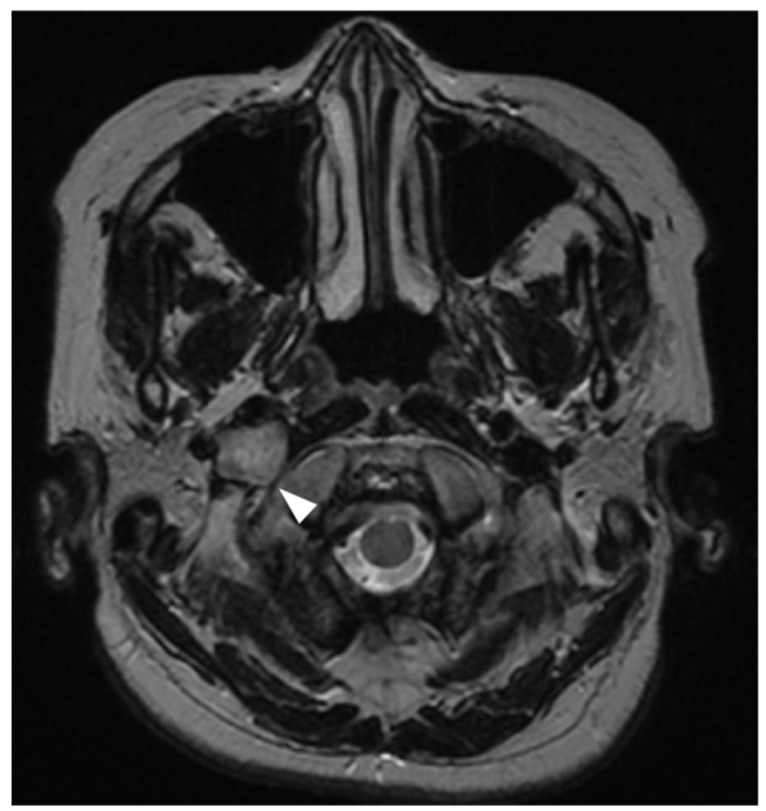
Axial T2-weighted magnetic resonance imaging demonstrating a 17 mm mass lesion located dorsal to the right internal carotid artery (arrow head).

A 62-year-old woman presented with dysarthria and right-sided tongue deviation with atrophy. MRI demonstrated a 17 mm mass lesion dorsal to the right internal carotid artery and adjacent to the right hypoglossal canal (Figure 1), leading to a presumptive diagnosis of hypoglossal schwannoma.

The patient elected conservative management with radiological follow-up. Over the following three years, progressive dysphagia and hoarseness developed, accompanied by atrophy of the right trapezius muscle. Follow-up imaging revealed tumor enlargement to 30 mm in diameter, prompting reconsideration of surgical treatment.

**Figure 2:**
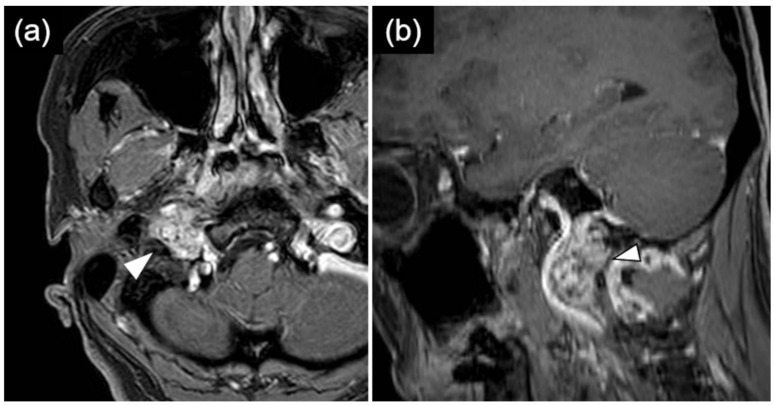
Contrast-enhanced T1-weighted magnetic resonance imaging demonstrating a 30 mm enhancing mass lesion located dorsal to the right internal carotid artery, extending extracranially and reaching the level of the first cervical vertebra (arrow head). Axial (**a**) and sagittal (**b**) views are shown.

**Figure 3:**
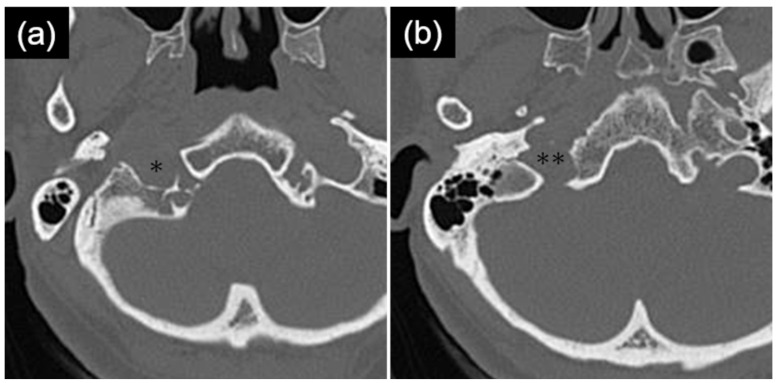
Thin-slice computed tomography with bone window settings. (**a**) Enlargement of the hypoglossal canal (*) is observed, most prominently along its lateral (extracranial) portion. (**b**) The enlarged hypoglossal canal is continuous with the expanded jugular foramen (**).

Preoperative MRI demonstrated extracranial extension of the tumor to the level of the first cervical vertebra (Figure 2a,b), and CT showed enlargement of the right hypoglossal canal (Figure 3a). Tumor resection via a transcondylar approach with opening of the hypoglossal canal was therefore planned.

**Figure 4:**
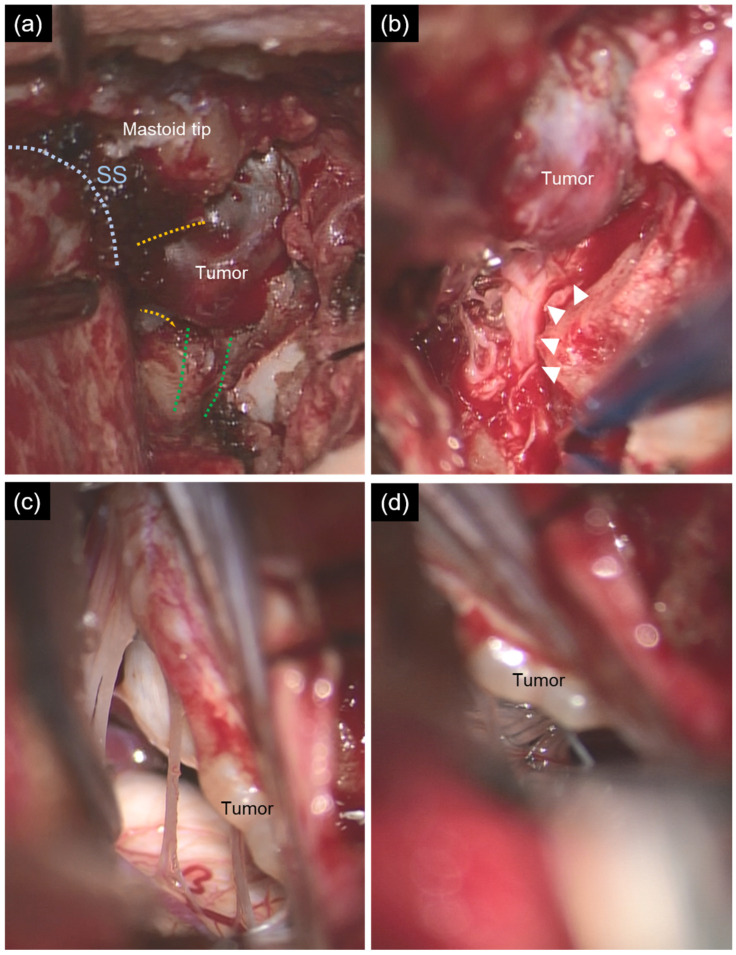
Intraoperative photographs. (**a**) Only a small tumor component was identified within the hypoglossal canal (green dashed lines), whereas the larger extracranial tumor was continuous with the jugular foramen (yellow dashed lines). SS, sigmoid sinus (blue dashed line). (**b**) Elevation of the tumor component within the hypoglossal canal revealed the compressed hypoglossal nerve (arrow heads). (**c**) Intradural exploration demonstrated a tumor arising from the accessory nerve, while the glossopharyngeal and vagus nerves were intact. (**d**) An intact hypoglossal nerve was identified.

Intraoperatively, only a small tumor component was identified within the hypoglossal canal, whereas the larger extracranial tumor was continuous with the jugular foramen (Figure 4a). Elevation of the tumor component within the hypoglossal canal revealed the compressed hypoglossal nerve without intraneural invasion (Figure 4b). Intradural inspection revealed a tumorous accessory nerve, while the glossopharyngeal, vagus, and hypoglossal nerves were intact. Based on these findings, the tumor was diagnosed as originating from the accessory nerve (Figure 4c,d).

**Figure 5:**
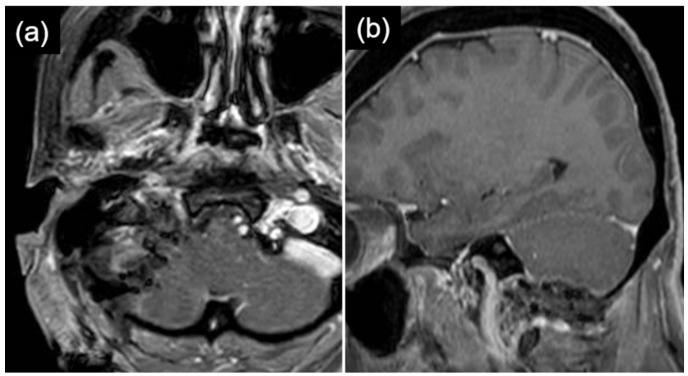
Postoperative contrast-enhanced T1-weighted magnetic resonance imaging demonstrating complete tumor resection. Axial (**a**) and sagittal (**b**) views are shown.

**Figure 6:**
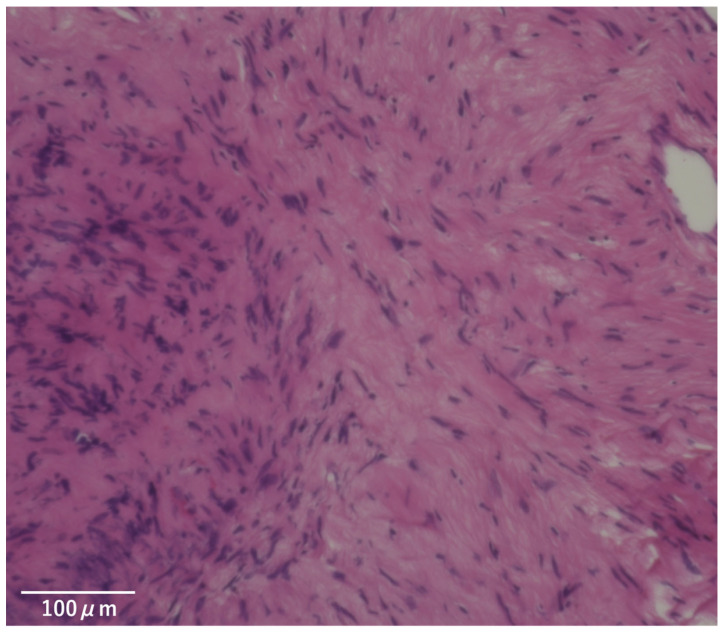
Histological examination demonstrating features typical of schwannoma, characterized by a biphasic tumor pattern with compact Antoni A areas showing nuclear palisading alternating with loosely arranged, hypocellular Antoni B areas.

Radiographically complete intracapsular resection of the tumor and its extracranial extension was achieved without complications (Figure 5). Histopathological examination confirmed schwannoma (Figure 6). At the 16-month follow-up, hoarseness and dysphagia had completely resolved. Mild rightward tongue deviation persisted, whereas tongue atrophy and sternocleidomastoid muscle atrophy remained. Trapezius muscle atrophy showed partial improvement over time. No delayed worsening of accessory nerve dysfunction was observed.

Importantly, retrospective review of the CT images revealed that the enlargement was confined to the extracranial portion of the hypoglossal canal and was continuous with the enlarged jugular foramen (Figure 3a,b). This finding serves as an important diagnostic clue for differentiating secondary hypoglossal canal involvement from primary hypoglossal canal tumors and highlights the critical neuroanatomical overlap between the jugular foramen and the hypoglossal canal.

## 4. Discussion

### 4.1. Clinical Characteristics of Intracranial Accessory Nerve Schwannomas

In this narrative review, we analyzed the clinical characteristics of intracranial accessory nerve schwannomas with particular emphasis on symptom distribution, temporal evolution of cranial nerve dysfunction, and the uncommon occurrence of hypoglossal nerve palsy. The analysis of the 57 reported cases demonstrated marked heterogeneity in clinical presentation, which was largely influenced by tumor location and growth pattern.

Consistent with our results, intracisternal tumors were reported more frequently than intrajugular tumors. Intracisternal accessory nerve schwannomas predominantly presented with symptoms related to posterior fossa compression, including headache, nausea/vomiting, gait ataxia, and ataxia. In contrast, intrajugular tumors were more commonly associated with lower cranial nerve dysfunction, such as hoarseness, dysphagia, shoulder weakness, and atrophy of the sternocleidomastoid and/or trapezius muscles. These findings underscore that the clinical manifestations of accessory nerve schwannomas are strongly dependent on tumor location.

Importantly, lower cranial nerve motor deficits were relatively uncommon at initial presentation but became increasingly apparent during the preoperative course, particularly in intrajugular tumors. This temporal progression, demonstrated in Table 4, indicates that early symptoms alone may not reflect the full extent of cranial nerve involvement and highlights the importance of longitudinal clinical assessment.

### 4.2. Mechanisms of Hypoglossal Nerve Palsy in Accessory Nerve Schwannomas

Hypoglossal nerve palsy is frequently observed in hypoglossal schwannomas [6,15,16,17], whereas it is rare in jugular foramen schwannomas, including accessory nerve schwannomas [19,20]. In the present review, hypoglossal nerve palsy was identified as an initial presenting symptom in three cases and as a preoperative finding in a total of seven cases.

The predominance of intrajugular tumors among cases with hypoglossal nerve palsy suggests a location-dependent mechanism. Anatomically, the lateral aspect of the jugular foramen is closely adjacent to the extracranial portion of the hypoglossal canal. Tumor extension from the jugular foramen toward the extracranial portion of the hypoglossal canal may therefore result in secondary compression of the hypoglossal nerve, even when the tumor does not originate from the hypoglossal nerve itself.

This mechanism is strongly supported by the illustrative case presented in this study. Retrospective bone-window CT demonstrated continuity between enlargement of the jugular foramen and expansion of the extracranial portion of the hypoglossal canal, while intraoperative findings confirmed hypoglossal nerve compression at the extracranial segment of the canal without intraneural invasion. Together, these observations provide a plausible anatomical explanation for hypoglossal nerve palsy in accessory nerve schwannomas.

In contrast, hypoglossal nerve palsy was rare in intracisternal tumors, likely because a larger tumor volume is required to exert sufficient mass effect on the hypoglossal nerve. In such cases, symptoms related to posterior fossa compression typically predominate.

### 4.3. Diagnostic Pitfalls and the Importance of Temporal Symptom Assessment

One of the key findings of this review is that the initial clinical presentation of accessory nerve schwannomas does not necessarily reflect the nerve of origin. This discrepancy was particularly evident in cases presenting with hypoglossal nerve palsy, which may lead to misdiagnosis as hypoglossal schwannoma based on clinical and radiological findings alone.

Temporal analysis revealed that dysfunction of the accessory nerve and other lower cranial nerves, including the hypoglossal nerve, frequently developed during the preoperative course rather than at symptom onset. Reliance on initial symptoms alone may therefore underestimate accessory nerve involvement and increase the risk of diagnostic error.

Accurate preoperative identification of the nerve of origin is clinically important, as it directly influences surgical planning, risk assessment, and patient counseling in skull base surgery. Detailed evaluation of skull base imaging, particularly assessment of the pattern and continuity of foraminal enlargement, is essential. Among cases presenting with hypoglossal nerve palsy, the most useful imaging clue suggesting accessory nerve schwannoma was enlargement of the jugular foramen, particularly when accompanied by secondary expansion of the extracranial portion of the hypoglossal canal. When continuity between these two enlarged foraminal structures was demonstrated, this finding favored secondary hypoglossal nerve compression by a jugular foramen tumor rather than a primary hypoglossal schwannoma. However, because this feature was documented only in selected cases, its absence does not exclude accessory nerve origin.

In contrast, primary hypoglossal schwannoma is more likely to be centered on the hypoglossal canal itself and to present more directly with isolated hypoglossal nerve dysfunction, particularly tongue deviation, tongue atrophy, and dysarthria. By comparison, accessory nerve schwannoma causing secondary hypoglossal nerve palsy may show predominant jugular foramen involvement together with coexisting or subsequently developing deficits of other lower cranial nerves, especially CN IX–XI. Recognition of these differences may improve the differential diagnosis between true hypoglossal schwannoma and accessory nerve schwannoma with secondary CN XII compression.

### 4.4. Surgical Outcomes and Clinical Implications

Surgical resection remains the primary treatment option for symptomatic or large intracranial accessory nerve schwannomas. In the present review, hypoglossal nerve function (CN XII) improved in six of seven cases and remained unchanged in one case, with no instances of postoperative worsening. These findings suggest that hypoglossal nerve palsy associated with accessory nerve schwannomas most likely reflects a reversible secondary compressive mechanism rather than irreversible nerve injury in the majority of reported cases.

In contrast, preservation of accessory nerve function (CN XI) represents a distinct surgical challenge, because the accessory nerve is the nerve of origin. Postoperative deterioration of accessory nerve function was reported in a small number of cases, underscoring the potential tradeoff between achieving adequate tumor resection and avoiding additional CN XI morbidity. Thus, while improvement in CN XII dysfunction may be expected when the deficit is caused by secondary compression, functional preservation of CN XI cannot be assumed even when resection is technically successful.

These different postoperative patterns have important implications for preoperative counseling. Patients should be informed not only of the potential for recovery of hypoglossal nerve function but also of the possibility of persistent or newly worsened accessory nerve dysfunction after surgery.

Although favorable outcomes with stereotactic radiosurgery have been reported, such treatments are generally reserved for small- to medium-sized tumors (median tumor volume: 2.6–4.9 cm^3^) or residual/recurrent lesions [48,60,61]. In large intrajugular tumors associated with progressive lower cranial nerve dysfunction, surgical intervention remains a crucial treatment strategy.

### 4.5. Limitations

This study has several limitations. First, it was conducted as a narrative review rather than a PRISMA-based systematic review. Second, the available evidence consisted mainly of retrospective case reports and small case series, which limits the strength of the conclusions. Publication and reporting bias may also have influenced the dataset. In addition, reporting of clinical symptoms, imaging findings, and outcomes was heterogeneous across studies. In particular, the distinction between initial and preoperative symptoms was not always uniformly described in the original reports, which may have affected the temporal analysis. Although the nerve of origin was identified primarily on the basis of intraoperative findings in most reports, the level of certainty may not have been entirely uniform across all historical cases. Long-term follow-up data were also limited. Finally, because the reviewed cases spanned a long period (1961–2025), changes in imaging quality, surgical practice, and reporting standards may have influenced the interpretation of the findings. Despite these limitations, this review provides a clinically useful synthesis of the currently available evidence on this rare entity.

## 5. Conclusions

Accessory nerve schwannomas with intracranial involvement or extension to the jugular foramen are rare tumors with heterogeneous clinical manifestations that appear to be strongly influenced by tumor location and growth pattern. Although lower cranial nerve dysfunction is common, the available literature suggests that hypoglossal nerve palsy—typically regarded as a hallmark of hypoglossal schwannoma—may also occur in accessory nerve schwannomas, most likely as a result of secondary compression caused by tumor extension from the jugular foramen toward the extracranial portion of the hypoglossal canal. This close anatomical relationship may represent an important source of diagnostic confusion.

The present review also suggests that the initial neurological presentation does not always indicate the true nerve of origin. Failure to recognize this potential mismatch may contribute to misclassification of skull base tumors and suboptimal surgical planning. Careful assessment of symptom progression, combined with meticulous skull base imaging—particularly evaluation of the continuity between jugular foramen enlargement and expansion of the extracranial hypoglossal canal when present—may assist in accurate preoperative diagnosis.

Recognition of these anatomical and clinical features may improve diagnostic accuracy, surgical strategy, and patient counseling in the management of this rare entity.

## Figures and Tables

**Table 1:** Clinical Characteristics of Reported Intracranial and Jugular Foramen Accessory Nerve Schwannomas.

No	Authors & Year	Sex	Age	Symptoms		Duration from Symptom Onset to Surgery	Location			Tumor Volume *	Surgical Approach	Preoperative CN Deficit	New Postoperative CN Deficit	Postoperative CN Recovery
				Initial	Preoperative		Julow et al. [11] Classification	JFS Classification	Details					
1	Ruberti et al., 1961 [22]	male	31	hearing loss	dysphagia, hoarseness, shoulder weakness, sternocleidomastoid and trapezius muscles atrophy, gait ataxia	8 m	Intrajugular type	Type A	Cerebellopontine angle with extension to the jugular foramen	-	Midline suboccipital approach	VIII, IX, X, XI	None	IX, X
2	Pluchino et al., 1975 [23]	female	42	headache	absent corneal reflexes, hearing loss, hoarseness, tongue atrophy, gait ataxia	2 y	Intrajugular type	Type D	Involving the medial aspect of the petrous bone and the occipital condyle	-	Lateral suboccipital approach	V, VIII, X, XII	None	V, VIII, X, XII
3	Pou-Serradell et al., 1978 [24]	male	56	hoarseness	hearing loss, dysphagia, shoulder weakness, shoulder pain, sternocleidomastoid and trapezius muscles atrophy, tongue atrophy	2 y	Intrajugular type	-	-	-	Lateral suboccipital approach	VIII, IX, X, XI, XII	None	VIII, IX, X, XII
4	Christoferson et al., 1982 [25]	female	24	trapezius muscle pain, arm and hand numbness, leg weakness	Clonus of the right ankle	1 y	Intracisternal type	Type A	Foramen magnum	13.1	Midline suboccipital approach	XI	None	None
5	Tohyama et al., 1982 [26]	male	52	burning sensation in his right forearm	sternocleidomastoid and trapezius muscles atrophy and weakness, bladder and rectal dysfunction	5 y	Intracisternal type	Type A	Foramen magnum	-	Midline suboccipital approach	-	-	-
6	Tsuchiya et al., 1982 [27]	male	18	nausea, vomiting, gait ataxia	left absence of corneal reflex, left abduction deficit	9 m	Intracisternal type	Type A	Cerebellopontine angle	-	Midline suboccipital approach	VI, VII	-	-
7	Julow et al., 1983 [11]	female	50	headache, neck pain	papilledema, sternocleidomastoid and trapezius weakness	3 y	Intracisternal type	Type A	Foramen magnum	48.0	Midline suboccipital approach	XI	None	XI
8	Julow et al., 1983 [11]	male	29	slight tetraparesis, dysmetria	sternocleidomastoid and trapezius muscles atrophy and weakness	4 y	Intracisternal type	Type A	Foramen magnum	-	Midline suboccipital approach with C1 laminectomy	XI	None	XI
9	Lesoin et al., 1984 [28]	male	23	hearing loss, gait ataxia, papilledema	None	-	Intracisternal type	Type A	Foramen magnum	-	Lateral suboccipital approach	VIII	-	-
10	Nishiura et al., 1984 [29]	male	52	burning sensation in his right forearm, numbness in both his hands	sternomastoid and trapezius atrophy, quadriparesis, bladder and rectal dysfunction	5 y	Intracisternal type	Type A	Foramen magnum	-	Midline suboccipital approach with C1–3 laminectomy	XI	None	None
11	Matsushima et al., 1985 [30]	male	51	nausea, vertigo, gait ataxia, taste disturbance	hearing loss, hyperesthesia of left body	15 m	Intracisternal type	Type A	Cerebellopontine angle	-	Lateral suboccipital approach	V, VII, VIII	IX, X, XI	-
12	Nakashima et al., 1988 [31]	male	62	hoarseness	hearing loss, dysphagia, trapezius weakness, sternocleidomastoid muscle atrophy	5 y	Intrajugular type	Type D	Jugular foramen tumor extending into the posterior fossa and extracranial space	-	Lateral suboccipital approach with intralabyrinthine approach	VII, IX, X, XI	-	-
13	Shiroyama et al., 1988 [32]	female	47	nausea, vomiting, gait ataxia	papilledema, dysphagia	2 m	Intracisternal type	Type A	Cerebellopontine angle	-	Lateral suboccipital approach	IX	-	-
14	Shiroyama et al., 1988 [32]	male	49	tinnitus	papilledema, fundus hemorrhage	10 m	Intracisternal type	Type A	Cerebellopontine angle	-	Midline suboccipital approach	None	-	-
15	Chang et al., 1990 [33]	male	32	headaches, nausea, vomiting	gait ataxia, papilledema	3 m	Intracisternal type	Type D	Foramen magnum	20.0	Lateral suboccipital approach with a C1-C2 laminectomy	None	None	XI
16	Iwasaki, et al., 1991 [10]	male	60	neck pain, dysphagia, hoarseness	shoulder weakness, sternomastoid and trapezius atrophy	several years	Intrajugular type	Type B	Jugular foramen	0.5	Lateral suboccipital approach	IX, X, XI	IX	X, XI
17	Fransen et al., 1992 [34]	female	27	headache, right upper extremity numbness	trapezius weakness, gait ataxia, papilledema	7 m	Intracisternal type	Type A	Cerebellomedullary	-	Midline suboccipital approach with C1 laminectomy	XI	None	XI
18	Sawada et al., 1992 [35]	male	40	dysphagia, hoarseness, shoulder weakness	sternomastoid and trapezius atrophy	8 y	Intrajugular type	Type B	Jugular foramen	-	Lateral suboccipital approach	IX, X, XI	None	XI
19	Aksik, 1993 [36]	-	-	spasmodic torticollis	None	-	Intracisternal type	Type A	-	0.03	Midline suboccipital approach with C1 laminectomy	XI	None	None
20	Aksik, 1993 [36]	-	-	spasmodic torticollis	None	-	Intracisternal type	Type A	-	0.03	Midline suboccipital approach with C1 laminectomy	XI	None	None
21	Lanotte et al., 1994 [37]	male	62	headache, nausea, vertigo, gait ataxia	None	3 m	Intracisternal type	Type A	Foramen magnum	11.2	Midline suboccipital approach with C1 laminectomy	None	XI	None
22	Tsukamoto et al., 1994 [9]	female	46	headache, diplopia, dysphagia, dysarthria, gait ataxia	hypesthesia of the left face, facial palsy	2 y	Intracisternal type	Type A	Cerebellopontine angle	0.06	Lateral suboccipital approach	V, VI, VII, IX, X	VIII	V, VI, VII, IX, X
23	Ortiz et al., 1995 [19]	male	76	headaches, nausea, vomiting, gait ataxia	sternomastoid and trapezius atrophy	1.5 m	Intrajugular type	Type D	Jugular foramen with extracranial extension	-	Lateral suboccipital approach	XI	-	-
24	Samii et al., 1995 [1]	male	43	facial prickling	None	-	Intrajugular type	Type A	-	-	Lateral suboccipital approach	V	None	V
25	Soo et al., 1995 [38]	male	54	headache, nausea	papilledema, gait ataxia	7 m	Intracisternal type	Type A	Foramen magnum extending to cerebellar vermis	-	Midline suboccipital approach with C1 laminectomy	None	None	None
26	Ohkawa et al., 1996 [39]	female	54	headache, nausea	papilledema, diplopia, vertigo, gait ataxia	7 m	Intracisternal type	Type A	Foramen magnum	-	Midline suboccipital approach with C1 laminectomy	-	None	-
27	Caputi et al., 1997 [40]	male	42	headache, nausea, vomiting	None	1 m	Intracisternal type	Type A	Foramen magnum	1.43	Midline suboccipital approach with C1 laminectomy	None	None	None
28	Saydam et al., 1997 [41]	female	52	hoarseness, shoulder weakness	dysphagia, trapezius atrophy, tongue deviation	1 y	Intrajugular type	Type C	Jugular foramen with extracranial extension	-	Transcervical-transmandibular approach	IX, X, XI, XII	None	IX, X, XI, XII
29	Kubota et al., 1998 [42]	female	40	-	headache, nausea, blurred vision, papilledema	-	Intracisternal type	Type A	Cerebellomedullary	-	Midline suboccipital approach with C1 laminectomy	None	None	None
30	Yoo et al., 1999 [21]	female	31	obscuration of vision	papilledema, afferent pupillary defect, abducens nerve palsy	10 m	Intrajugular type	Type A	-	-	Lateral suboccipital approach	VI	None	-
31	Yoo et al., 1999 [21]	female	40	tinnitus	shoulder weakness, LCN dysfunction (details unspecified), nystagmus, gait ataxia	1 y	Intrajugular type	Type D	-	-	Lateral suboccipital approach	IX, X, XI	None	-
32	Lee et al., 2001 [3]	female	48	hemifacial spasm	hearing loss, hoarseness, dysphagia, truncal ataxia	7 y	Intrajugular type	Type D	-	-	Petrooccipital transsigmoid approach	VII, VIII, IX, X	-	-
33	Sarma et al., 2002 [6]	-	-	-	a typical jugular foramen syndrome (details unspecified)	-	Intrajugular type	Type B	-	-	Retrosigmoid-extreme lateral approach	IX, X, XI	None	IX, X, XI
34	Tatebayashi et al., 2003 [43]	female	46	headache, neck pain, shoulder pain	nausea, vomiting, right upper extremity numbness	1 y	Intracisternal type	Type A	Foramen magnum and medulla oblongata.	31.5	Midline suboccipital approach with C1 laminectomy	None	None	None
35	Kurokawa et al., 2004 [12]	male	50	neck pain	None	2 m	Intracisternal type	Type A	Extending from the foramen magnum to the fourth ventricle	21.4	Midline suboccipital approach with C1 laminectomy	None	None	None
36	Takumi et al., 2005 [44]	male	35	headache, neck pain	motor weakness in the bilateral distal upper limbs numbness in the right upper limb	6 m	Intracisternal type	Type A	Foramen magnum	-	The postero-lateral approach	None	None	None
37	Wilson et al., 2005 [45]	-	-	tongue deviation	None	-	Intrajugular type	Type B	Jugular foramen with extracranial extension	>13.5 **	Transcondylar approach	XII	XI	XII
38	Wilson et al., 2005 [45]	-	-	-	dysphagia, hoarseness, tongue deviation	-	Intrajugular type	Type D	Jugular foramen tumor extending into the posterior fossa and extracranial space	>13.5 **	Infratemporal approach with partial VII mobilization	IX, X, XII	None	None
39	Jung et al., 2006 [46]	female	70	headache, neck pain	None	5 y	Intracisternal type	Type A	Cerebellomedullary	3.2 × 2.0	Midline suboccipital approach	None	None	None
40	Chibbaro et al., 2009 [47]	female	42	-	headache, vertigo, hoarseness	-	Intrajugular type	Type D	-	-	Juxtacondylar approach	X	-	X
41	Fukuda et al., 2009 [48]	male	47	-	-	-	Intrajugular type	Type D	-	-	Lateral suboccipital approach with transjugular approach	IX, X, XI, XII	-	-
42	Sadatomo et al., 2010 [49]	female	48	headache	None	-	Intracisternal type	Type A	Cerebellomedullary	3.75	Lateral suboccipital approach	None	None	None
43	Cavalcanti et al., 2011 [50]	female	47	hoarseness	None	-	Intrajugular type	Type B	Cerebellopontine angle and jugular foramen	-	Lateral suboccipital approach with transjugular approach	X	None	X
44	Chowdhury et al., 2014 [51]	male	38	-	neck pain, quadriparesis, sternomastoid and trapezius weakness	-	Intracisternal type	Type A	Foramen magnum and posterior fossa	3.0 × 4.0	Lateral suboccipital approach with C1 laminectomy	XI	None	XI
45	Chowdhury et al., 2014 [51]	female	52	-	headache, neck pain, quadriparesis, sternomastoid and trapezius atrophy	-	Intracisternal type	Type A	Posterior fossa and foramen magnum with extracranial extension	3.0 × 3.0	Midline suboccipital approach with C1 laminectomy	XI	None	None
46	Jin et al., 2014 [7]	female	58	dizziness, hoarseness	left side of body numbness	2 m	Intracisternal type	Type A	Cerebellomedullary	14.7	Lateral suboccipital approach with C1 laminectomy	X	None	X
47	Nowak et al., 2014 [20]	female	25	shoulder weakness	dysphagia, hoarseness	-	Intrajugular type	Type D	Jugular foramen with extracranial extension	-	Lateral suboccipital approach with C1 laminectomy	IX, X, XI	VII, IX, X	None
48	Krishnan et al. 2015 [52]	male	22	headache, obscuration of vision	gait ataxia, papilledema	6 m	Intracisternal type	Type A	Extending from the foramen magnum to the fourth ventricle	-	Midline suboccipital approach with C1 laminectomy	None	None	None
49	Richard et al., 2017 [13]	female	52	dizziness, hoarseness, tongue deviation	None	6 m	Intracisternal type	Type A	Foramen of Luschka extending to foramen magnum	21.9	Lateral suboccipital approach with C1 laminectomy	X, XII	None	XII
50	Antoniades et al., 2019 [53]	male	43	-	headache, neck pain, dizziness, tinnitus	-	Intracisternal type	Type A	Foramen magnum	1.6 × 2.	Midline suboccipital approach with C1 laminectomy	None	None	None
51	Antoniades et al., 2019 [53]	female	36	None	None	-	Intracisternal type	Type A	Foramen magnum	1.1 × 1.0	Midline suboccipital approach with C1 laminectomy	None	None	None
52	Antoniades et al., 2019 [53]	male	45	-	headache, neck pain,	-	Intracisternal type	Type A	Foramen magnum	1.2 × 1.0	Midline suboccipital approach with C1 laminectomy	None	None	None
53	Watanabe et al., 2019 [54]	male	68	-	dizziness numbness in the right upper limb	-	Intracisternal type	Type A	Foramen magnum	-	Midline suboccipital approach with C1 laminectomy	None	None	None
54	Yan et al., 2020 [14]	female	62	headache	None	1 m	Intracisternal type	Type A	Cerebellomedullary	-	Midline suboccipital approach with C1 laminectomy	None	XI	None
55	Paula et al., 2022 [55]	male	61	dysmetria, gait ataxia	vomiting	3 m	Intracisternal type	Type A	Cerebellomedullary	29.9	Extended far lateral retrosigmoid approach with C1 hemilaminectomy	None	X	None
56	Lasica et al., 2024 [56]	female	60	right-sided weakness	None	3 m	Intracisternal type	Type A	Foramen of Luschka extending to the foramen magnum	-	Far lateral suboccipital approach with C1 hemilaminectomy	None	None	None
57	Naser et al., 2025 [57]	female	61	headache	None	-	Intracisternal type	Type A	Foramen magnum extending rostrally to the fourth ventricle	-	Midline suboccipital approach with C1 laminectomy	None	None	None
58	Present case	female	62	tongue deviation and atrophy, dysarthria	dysphagia, hoarseness, trapezius muscle atrophy	3 y	Intrajugular type	Type B	Jugular foramen with extracranial extension (partly into the hypoglossal canal)	10.3	Transcondylar approach	IX, X, XI, XII	None	IX, X, XII

- indicates data not reported in the original publication. * When all three tumor dimensions (a, b, and c) were available, tumor volume was estimated using the ellipsoid formula (a × b × c/2, cm^3^). When only two dimensions were reported, the corresponding lengths (cm) were presented without volume estimation. ** In the report by Wilson et al., tumor volume was not explicitly described; however, the minimum reported tumor diameter was 3 cm [45]. Assuming a spherical tumor with a diameter of 3 cm, the estimated tumor volume was at least 13.5 cm^3^. CN, cranial nerve; JFS, jugular foramen schwannoma; LCN, lower cranial nerve; m, month; y, year.

**Table 2:** Frequency of Initial Symptoms in Intracranial Accessory Nerve Schwannomas.

Initial Symptom	Intrajugular Type, *n* (%)	Intracisternal Type, *n* (%)	Total, *n* (%)
Headache	3 (15.0)	13 (35.1)	16 (28.1)
Nausea/Vomiting	1 (5.0)	8 (21.6)	9 (15.8)
Hoarseness (CN X)	6 (30.0)	2 (5.4)	8 (14.0)
Gait ataxia	1 (5.0)	6 (16.2)	7 (12.3)
Neck pain	1 (5.0)	5 (13.5)	6 (10.7)
Shoulder weakness (CN XI)	4 (20.0)	0 (0.0)	4 (7.0)
Dizziness/Vertigo	0 (0.0)	3 (8.1)	3 (5.3)
Tongue deviation/atrophy (CN XII)	2 (10.0)	1 (2.7)	3 (5.3)
Visual disturbance	1 (5.0)	1 (2.7)	2 (3.5)
Hearing loss (CN VIII)	1 (5.0)	1 (2.7)	2 (3.5)
Dysphagia (CN IX)	2 (10.0)	0 (0.0)	2 (3.5)
Shoulder pain (CN IX)	0 (0.0)	2 (5.4)	2 (3.5)
Spasmodic torticollis	0 (0.0)	2 (5.4)	2 (3.5)
Dysarthria (CN XII)	1 (5.0)	0 (0.0)	1 (1.8)

Tsukamoto et al. were excluded because the reported symptoms were mainly attributable to a concomitant giant meningioma [9]; therefore, the denominator for Table 2, Table 3 and Table 4 is 57 cases. Percentages were calculated based on 20 intrajugular, 37 intracisternal, and 57 total cases. CN, cranial nerve.

**Table 3:** Frequency of Initial and Preoperative Symptoms in Intracranial Accessory Nerve Schwannomas.

Symptom	Intrajugular Type, *n* (%)	Intracisternal Type, *n* (%)	Total, *n* (%)
Headache	3 (15.0)	18 (48.6)	21 (36.8)
Hoarseness (CN X)	13 (65.0)	2 (5.4)	15 (26.3)
Gait ataxia	4 (20.0)	11 (29.7)	15 (26.3)
Nausea/Vomiting	1 (5.0)	11 (29.7)	12 (21.1)
SCM and/or trapezius muscle atrophy (CN XI)	8 (40.0)	4 (10.8)	12 (21.1)
Shoulder weakness (CN XI)	7 (35.0)	5 (13.5)	12 (21.1)
Dysphagia (CN IX)	9 (45.0)	1 (2.7)	10 (17.5)
Neck pain	1 (5.0)	9 (24.3)	10 (17.5)
Hearing loss (CNVIII)	5 (25.0)	2 (5.4)	7 (12.3)
Tongue deviation/atrophy (CN XII)	6 (30.0)	1 (2.7)	7 (12.3)
Dizziness/Vertigo	1 (5.0)	6 (16.2)	7 (12.3)
Visual disturbance	1 (5.0)	3 (8.1)	4 (7.0)
Shoulder pain (CN XI)	1 (5.0)	2 (5.4)	3 (5.3)
Spasmodic torticollis	0 (0.0)	2 (5.4)	2 (3.5)
Abduction deficit (CN VI)	0 (0.0)	1 (2.7)	1 (1.8)
Absence of corneal reflex (CN VII)	0 (0.0)	1 (2.7)	1 (1.8)
Dysarthria (CN XII)	1 (5.0)	0 (0.0)	1 (1.8)

Tsukamoto et al. were excluded because the reported symptoms were mainly attributable to a concomitant giant meningioma [9]; therefore, the denominator for Table 2, Table 3 and Table 4 is 57 cases. Percentages were calculated based on 20 intrajugular, 37 intracisternal, and 57 total cases. CN, cranial nerve; SCM, sternocleidomastoid muscle.

**Table 4:** Evolution of Cranial Nerve Symptoms in Intracranial Accessory Nerve Schwannomas According to Tumor Location.

Symptom	Intrajugular Type, *n* (%)		Intracisternal Type, *n* (%)		Total, *n* (%)
	Initial	Preoperative	Initial	Preoperative	
Abduction deficit (CN VI)	0 (0.0)	0 (0.0)	0 (0.0)	1 (2.7)	1 (1.8)
Absence of corneal reflex (CN VII)	0 (0.0)	0 (0.0)	0 (0.0)	1 (2.7)	1 (1.8)
Hearing loss (CNVIII)	1 (5.0)	4 (20.0)	1 (2.7)	1 (2.7)	7 (12.3)
Dysphagia (CN IX)	2 (10.0)	7 (35.0)	0 (0.0)	1 (2.7)	10 (17.5)
Hoarseness (CN X)	6 (30.0)	7 (35.0)	2 (5.4)	0 (0.0)	15 (26.3)
Shoulder weakness (CN XI)	4 (20.0)	4 (20.0)	0 (0.0)	4 (10.8)	12 (21.1)
SCM/Trapezius muscle atrophy (CN XI)	0 (0.0)	8 (40.0)	0 (0.0)	4 (10.8)	12 (21.1)
Shoulder pain (CN XI)	0 (0.0)	1 (5.0)	2 (5.4)	0 (0.0)	3 (5.3)
Tongue deviation/atrophy (CN XII)	3 (15.0)	3 (15.0)	1 (2.7)	0 (0.0)	7 (12.3)
Dysarthria (CN XII)	1 (5.0)	0 (0.0)	0 (0.0)	0 (0.0)	1 (1.8)

Tsukamoto et al. were excluded because the reported symptoms were mainly attributable to a concomitant giant meningioma [9]; therefore, the denominator for Table 2, Table 3 and Table 4 is 57 cases. Percentages were calculated based on 20 intrajugular, 37 intracisternal, and 57 total cases. CN, cranial nerve; SCM, sternocleidomastoid muscle.

## Data Availability

The original contributions presented in this study are included in the article. Further inquiries can be directed to the corresponding author.

## Abbreviations

The following abbreviations are used in this manuscript:

CN	cranial nerve
CT	computed tomography
JFS classification	jugular foramen schwannoma classification
MRI	magnetic resonance imaging
